# Prospective evaluation of antral lesion size of the 31-mm size of a novel size-adjustable cryoballoon: Results of the BETTER-FIT study

**DOI:** 10.1016/j.hroo.2025.01.013

**Published:** 2025-03-22

**Authors:** Nick van Boven, Rohit Bhagwandien, Sip A. Wijchers, Mark Hoogendijk, Bakhtawar Khan Mahmoodi, Sing-Chien Yap

**Affiliations:** 1 Department of Cardiology, Thoraxcenter, Cardiovascular Institute, Erasmus Medical Center, Rotterdam, The Netherlands; 2 Department of Cardiology, Franciscus Gasthuis and Vlietland, Rotterdam, The Netherlands

**Keywords:** Atrial fibrillation, Cryoballoon ablation, Antral lesion size, High-definition mapping, Pulmonary vein isolation

## Abstract

**Background:**

A novel size-adjustable cryoballoon can deliver cryotherapy with a 28- or 31-mm balloon size. However, data on antral lesion size with the 31-mm balloon size are scarce.

**Objective:**

The purpose of this study was to evaluate the antral lesion size of cryoablation with the 31-mm balloon size.

**Methods:**

This prospective single-center study included patients with paroxysmal atrial fibrillation undergoing first-time pulmonary vein isolation (PVI). All pulmonary veins (PVs) were first ablated with the 31-mm balloon size. The 28-mm balloon size was only used as bailout. Pre- and postablation left atrial ultrahigh-definition mapping was performed to assess the antral lesion area. Secondary outcome measures were procedural efficacy including balloon occlusion grade.

**Results:**

Complete PVI was achieved in all 80 PVs in 20 patients (mean age 59.7 ± 10.7 years, 75% male). More than one-third of the posterior wall was ablated (35.4% ± 13.8%), and the isolated surface area was 68.7% ± 8.5%. Lateral and septal circumferential antral lesion areas were 12.1 ± 2.0 cm^2^ and 19.1 ± 4.7 cm^2^, respectively. One patient demonstrated inadvertent overlap of the antral lesions on the roof. There was a trend toward lower complete balloon occlusion in the right superior PV with the 31-mm balloon size in comparison to the 28-mm size (75% and 90%, *P* = .08).

**Conclusion:**

Cryoablation with the 31-mm size of a novel size-adjustable cryoballoon results in a large antral lesion. In small atria there is the potential for leaving a small nonablated corridor on the roof when using the 31-mm balloon in both superior PVs, which may be proarrhythmogenic.

**Key Findings**

▪ Ablation with the 31-mm balloon size of a size-adjustable cryoballoon creates large homogeneous antral lesions.
▪ There is the risk of proximity of opposite antral lesions on the roof creating a proarrhythmogenic substrate.
▪ There is a tendency toward suboptimal occlusion of the right inferior pulmonary vein with a bigger balloon size.

## Introduction

Pulmonary vein isolation (PVI) is a well-established therapy for the treatment of symptomatic atrial fibrillation (AF).^1^^,^^2^ PVI using a cryoballoon (CB) has been shown to be as safe and effective as radiofrequency (RF) ablation, but it has the advantage of shorter procedural times and less operator dependency.^3^, ^4^, ^5^, ^6^ In 2020, a novel 28-mm CB (POLARx, Boston Scientific, Marlborough, MA) was introduced in Europe. The main characteristic of this novel CB is that it operates with a constant lower inner balloon pressure that allows it to maintain a consistent 28-mm size throughout inflation and ablation. In 2023, a second generation of this novel CB (POLARx FIT, Boston Scientific) that allows cryoablation at 2 balloon sizes (28 or 31 mm) became available on the market. Several clinical studies have demonstrated the safety and effectiveness of the novel 28-mm CB with a high freedom of atrial arrhythmia recurrence,^7^, ^8^, ^9^, ^10^, ^11^, ^12^ but clinical data on the novel size-adjustable CB are limited.^12^, ^13^, ^14^, ^15^, ^16^, ^17^, ^18^, ^19^ Theoretically, a larger balloon size results in a larger antral lesion size and this has been supported by computer modeling and case series.^20^ A larger antral lesion size potentially may be beneficial in reducing atrial arrhythmia recurrence and may reduce the risk of phrenic nerve palsy (PNP) by a more antral balloon position.^21^ The aim of the current study was to evaluate the antral lesion size of the 31-mm balloon size of the novel size-adjustable CB using ultrahigh-density (UHDx) mapping in patients with paroxysmal AF undergoing first-time PVI. Secondary endpoints were the rate of complete pulmonary vein (PV) occlusion and single-shot success of the 31-mm CB size.

## Methods

### Study design and patient population

BETTER-FIT (Effect of a BiggEr Cryoballoon on The Total antral lesion size: Evaluation of POLARx FIT; ClinicalTrials.gov Identifier: NCT05881733) was an investigator-initiated, prospective, single-center, single-arm study, conducted in the Erasmus MC, Rotterdam, The Netherlands. Adult patients (≥18 years) were eligible for inclusion if they had symptomatic paroxysmal AF and were indicated for PVI according to the 2020 European Society of Cardiology guidelines.^22^ Patients were excluded if they had a contraindication for an AF ablation or anticoagulation; a history of previous left atrial (LA) ablation or surgical treatment of atrial tachyarrhythmias; AF secondary to reversible causes; structural heart disease; and common ostium PV >24 mm shown by preprocedural computed tomographic scan.

### Ethics statements

All patients who met the enrollment criteria, signed the consent, and underwent the index procedure were followed for up to 12 months after the procedure. The study was approved by the medical ethics committee of the Erasmus MC (MEC-2023-0325) and was conducted in accordance with the Declaration of Helsinki.

### Preablation workflow

**Figure 1:**
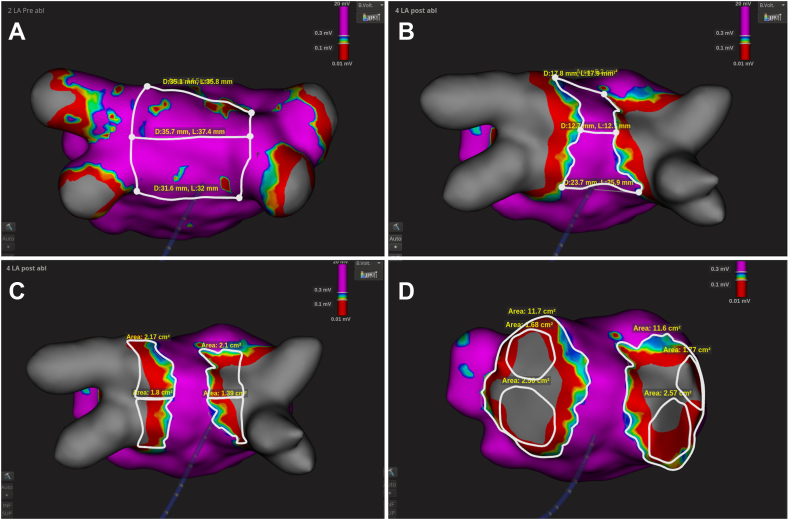
Overview of measurements pre- and postablation. **A:** Preablation measurements consisted of posterior wall surface area, and superior, middle, and inferior lines. **B:** Postablation measurements consisted of unablated posterior wall surface area, and superior, middle, and inferior lines using a cutoff value of 0.3 mV. **C:** Postablation measurements focusing on posterior antral lesion area at the level of each pulmonary vein. **D:** Postablation measurements focusing on antral lesion area on each side and the pulmonary vein ostia area. Note that the pulmonary veins were deleted to prevent overestimation of the antral lesion area.

All patients were on oral anticoagulation for at least 4 weeks before the procedure, and anticoagulation was not interrupted on the day of the procedure. Ablation was performed with patients under deep sedation using propofol and remifentanil. Before the start of the procedure, LA intracardiac thrombus was ruled out by transesophageal echocardiography. Femoral vein punctures were performed under ultrasound guidance. After placement of 2 short introducer sheaths (8F and 6F), a bolus of intravenous heparin (5000 IU) was administered. A steerable decapolar diagnostic catheter was initially placed in the coronary sinus (for pacing during vagal response and as a reference catheter during mapping). During right-sided cryoablation, the decapolar catheter was placed in the superior vena cava for phrenic nerve pacing. Transseptal puncture was performed using an SL1-sheath (Swartz, Abbott, Abbott Park, IL) and a transseptal needle (AcQCross, Medtronic, Minneapolis, MN [n = 18] or NRG transseptal needle, Bayliss Medical, Rouyn-Noranda, Canada [n = 2]) under guidance by transesophageal or intracardiac echocardiography. After transseptal puncture, another bolus of intravenous heparin (5000 IU) was administered to achieve a target activated clotting time >300 seconds. The SL1 sheath was replaced by the POLARSHEATH (Boston Scientific). Preablation 3-dimensional UHDx LA mapping was performed using a 64-pole mini-basket mapping catheter (INTELLAMAP ORION, Boston Scientific) and the RHYTHMIA HDx mapping system (Boston Scientific). Mapping was preferentially performed in sinus rhythm with prior cardioversion of AF if required. LA voltage maps were constructed with special point density in the antra of PVs with scar cutoff <0.3 mV.^23^^,^^24^ The following measurements were made on the preablation map: (1) left atrial posterior wall (LAPW) surface area; and (2) superior, middle, and inferior lines of the LAPW (Figure 1A). The superior line is defined as the distance between the superior part of the left superior pulmonary vein (LSPV) and right superior pulmonary vein (RSPV) (at the level of the roof). The middle line is defined as the distance between the left and right posterior carinas. The inferior line is defined as the distance between the inferior part of the left inferior pulmonary vein (LIPV) and right inferior pulmonary vein (RIPV).

### Ablation procedure

After preablation UHDx mapping, the CB catheter was inserted in the LA. First, we determined the best possible PV occlusion per PV using the 28 and 31 mm, sequentially. Using the POLARMAP circular mapping catheter (Boston Scientific) in the target PV, the CB is initially inflated to the standard 28-mm size. The most optimal PV occlusion with the 28-mm balloon size was achieved per target PV, and the grade of PV occlusion was determined using semiquantitative grading (1 = rapid outflow of contrast medium from the PV to 4 = complete contrast retention with no observable leak).^25^ Thereafter, the balloon was increased in size to 31 mm, and the best possible PV occlusion was achieved and graded again. After optimal PV occlusion with the 31-mm balloon size, a single cryoapplication was delivered. A 3-minute freeze cycle was used if the time to isolation was <60 seconds; otherwise, a 4-minute freeze cycle was used. The cryoapplication was prematurely stopped when the inner balloon temperature was –69°C. If PVI was not achieved after the first attempt, subsequent cryoapplications (3 or 4 minutes depending on time to isolation) were delivered until isolation. The balloon size during subsequent applications was adjusted according to the preferences of the operator.

The usual order of targeted PVs was LSPV, LIPV, RIPV, and finally RSPV. During right-sided PV ablation, high-output right phrenic nerve stimulation was performed using the decapolar catheter in the transition area of the right subclavian vein to superior vena cava. Diaphragmatic excursion was checked by a combination of manual palpation and use of the diaphragmatic movement sensor. Whenever the diaphragmatic excursions decreased, cryoablation was immediately terminated (double stop). Electrical isolation of a PV was demonstrated by entrance and exit block using the POLARMAP™ circular mapping catheter. Entrance block was defined as the absence of local potentials inside the vein. Exit block was defined as absence of atrial capture while pacing from inside the vein with the POLARMAP™ circular mapping catheter. Complete PVI was defined as electrical isolation of all PVs.

### Postablation workflow and measurements

**Figure 2:**
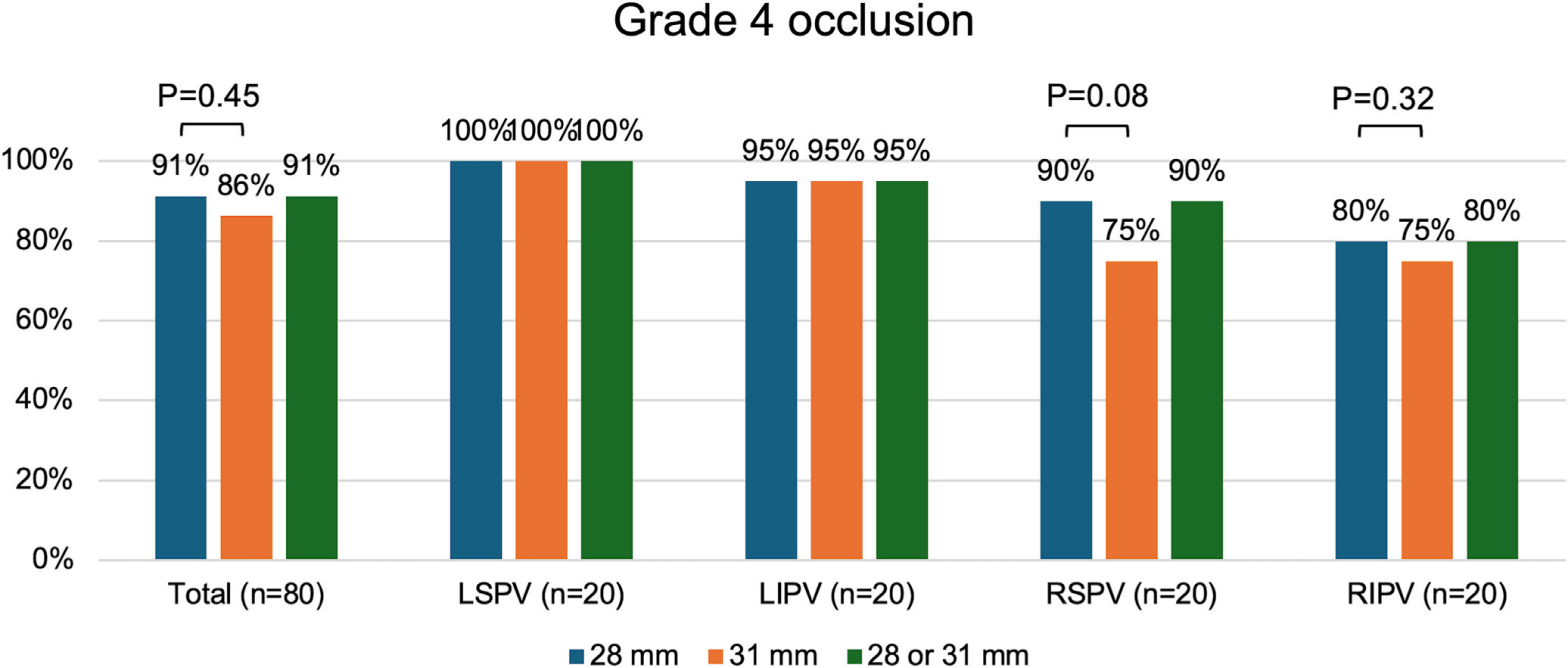
Grade 4 occlusion grade between the 28-mm and 31-mm cryoballoon size of the different pulmonary veins. There was a trend toward lower grade 4 occlusion in the right superior pulmonary vein (RSPV) with the 31-mm balloon size in comparison to the 28-mm balloon size (*P* = .08). *Green bar* denotes the grade 4 occlusion grade with either the 28- or 31-mm balloon size. Note there was no increase in grade 4 occlusion grade with both sizes in comparison to the 28-mm size only. LIPV = left inferior pulmonary vein; LSPV = left superior pulmonary vein; RIPV = right inferior pulmonary vein.

**Figure 3:**
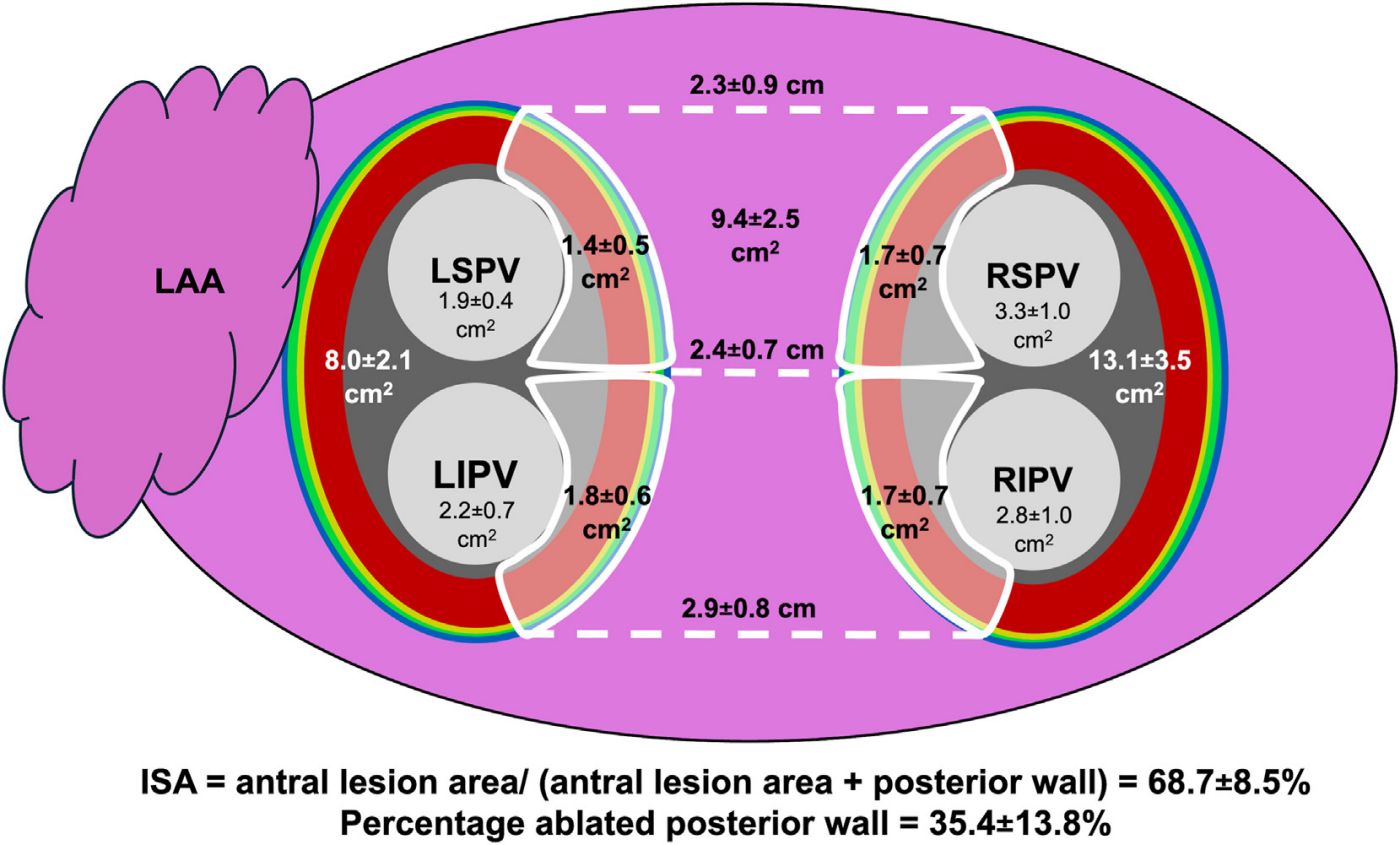
Summary of the characteristics of antral lesion size after ablation with the 31-mm balloon size. The nonablated posterior wall forms a trapezium with the smallest dimensions at the superior line (2.3 ± 0.9 cm) and the longest dimensions at the inferior line (2.9 ± 0.8 cm). Approximately one-third of the posterior wall is ablated (35.4% ± 13.8%). LAA = left atrial appendage; other abbreviations as in Figure 2.

**Figure 4:**
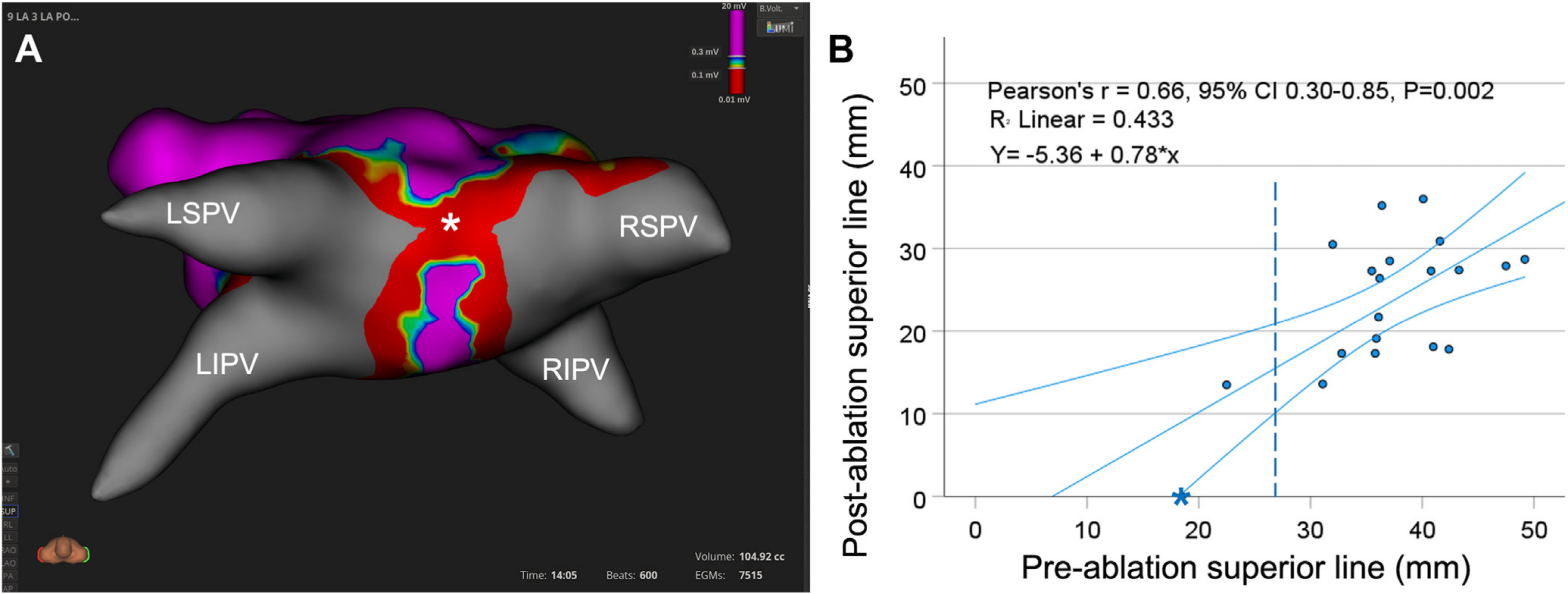
**A:** Inadvertent roof line in a study patient. **B:** Correlation between preablation superior line and postablation superior line. Pearson correlation = 0.66 (*P* = .002). The lower boundary of the 95% confidence interval transects the 10-mm postablation superior line at 27 mm *(dashed line). Asterisks* denote the patient with postablation inadvertent roof line. Abbreviations as in Figure 2.

After complete PVI was achieved, a repeat UHDx LA map was created using the same settings as previously described. If there was an early reconnected PV, this PV was again ablated with the CB. The following measurements were made with the final UHDx LA map in which all PVs were isolated: (1) the unablated LAPW surface area (Figure 1B); (2) the distance between the ipsilateral, antral levels of isolation at superior, middle, and inferior latitudes of the LAPW at the same level as the preablation measurements (Figure 1B); (3) the posterior antral lesion area of each PV bordered by the PV ostium, carina, and low-voltage area (Figure 1C); (4) total area of the circular antral lesion area surrounding each ipsilateral PV pair (Figure 1D); and (5) surface area of each PV ostium, where PV ostium was defined as the maximal inflection between the PV and the LA wall (Figure 1D). The true antral lesion area was assessed by the difference between the total circular antral lesion area minus the PV ostium areas.

### Follow-up

All patients underwent follow-up at our outpatient clinic at 3, 6, and 12 months after the index procedure with 24-hour Holter monitoring. In case of recurrent atrial tachyarrhythmia, the decision to perform a repeat ablation procedure was made at the discretion of the treating physician.

### Statistical analysis

Continuous data are given as mean ± SD or median [interquartile range]. Continuous data were analyzed using the Student *t* test or Mann–Whitney *U* test when appropriate. Categorical data were compared using the χ^2^ test or Fisher exact test when appropriate. The primary outcome was the extent of the antral lesion size, which was described using several measurements including the relative proportion (expressed as percentage) of the posterior wall area that was ablated; the posterior antral lesion surface area per PV; the septal and lateral true antral lesion area; and the isolated surface area (ISA). ISA was defined as the proportion (expressed as percentage) of the combined septal and lateral true antral lesion area relative to the combined septal and lateral true antral lesion area plus the unablated posterior wall surface area. Secondary study endpoints were the proportion of grade 4 occlusion with the 28-mm and 31-mm balloon size and the single-shot success rate of the 31-mm balloon size. The difference in the proportion of grade 4 occlusion between the 28-mm and 31-mm balloon sizes was compared using the McNemar test for paired nominal data.

## Results

### Baseline and procedural characteristics

A total of 20 patients were included in the study between October 2023 and April 2024. Baseline and procedural characteristics are given in Table 1. Mean age of the study population was 59.7 ± 10.7 years, and 75% of the patients was male. LA volume index was 32.4 ± 9.5 mL/m^2^, and all patients had normal left ventricular function. All patients used a direct acting oral anticoagulant and at least 1 antiarrhythmic drug. All patients were in sinus rhythm at the start of the procedure, and complete PVI could be achieved in all patients. Mean procedural time (including pre- and postablation mapping) was 87.0 ± 18.9 minutes, and median fluoroscopy time was 11.9 [10.4–15.9] minutes.

**Table 1:** Baseline and procedural characteristics (N = 20)

Baseline characteristics	
Age, y	59.7 ± 10.7
Male sex	15 (75)
Paroxysmal AF	20 (100)
EHRA classification	
1	2 (10)
2a	7 (35)
2b	9 (45)
3	2 (10)
Congestive heart failure	0 (0)
Hypertension	10 (50)
Diabetes	2 (10)
History of stroke or TIA	2 (10)
Valvular heart disease	0 (0)
Coronary artery disease	1 (5)
OSAS	2 (10)
BMI, kg/m^2^	28.4 ± 3.9
CH_2_ADS_2_-VASc score	1 [1–2.5]
0	3 (15)
1	8 (40)
2	4 (20)
3	4 (20)
4	1 (5)
LAVI, mL/m^2^	32.4 ± 9.5
LV function	
Normal (50%–70%)	20 (100)
DOAC	20 (100)
Antiarrhythmic drugs	20 (100)
Flecainide	8 (40)
Disopyramide	1 (5)
Beta-blockers	10 (50)
Sotalol	7 (35)
Amiodarone	1 (5)
Verapamil	2 (10)
Diltiazem	1 (5)
Digoxin	2 (10)
ARB	2 (10)
ACEI	3 (15)
MRA	1 (5)
Procedural characteristics	
Baseline rhythm	
Sinus rhythm	20 (100)
Procedural time, min	87.0 ± 18.9
Balloon in body time, min	36 [29–45]
Fluoroscopy time, min	11.9 [10.4–15.9]
Dose area product, cGycm^2^	684 [460–1152]
Radiation dose, mGy	62 [40–108]
No. of CBA applications	4.5 [4–5.5]
Total duration of CBA, s	911 [780–1050]
Complete PVI	20 (100)

Continuous values given as mean ± SD or median [first–third quartile]. Other values given as n (%).

ACEI = angiotensin-converting enzyme inhibitor; AF = atrial fibrillation; ARB = angiotensin receptor blocker; BMI = body mass index; CBA = cryoballoon ablation; DOAC = direct acting oral anticoagulant; EHRA = European Heart Rhythm Association; LAVI = left atrial volume index; LV = left ventricle; MRA = mineralocorticoid receptor antagonist; OSAS = obstructive sleep apnea syndrome; PVI = pulmonary vein isolation; TIA = transient ischemic attack.

### CB ablation metrics

CB ablation metrics are given in Table 2. A cryoapplication with the 31-mm balloon size was used in all PVs in all patients, except for the RIPV in 1 patient because of lack of occlusion. The single freeze ablation rate was high on the PV level (63/80 PVs [79%]) and patient level (10/20 patients [50%]). Median total number of CB applications was 4.5 [4–5.5]. There was a need for the 28-mm balloon size in only 22 of 103 CB applications (21%), most commonly in the superior PVs (77%).

**Table 2:** Cryoballoon metrics

Variable	LSPV	LIPV	RSPV	RIPV
Median no. of CBA applications	1 [1–2]	1 [1–1]	1 [1–2]	1 [1–1]
Duration of CBA, s	180 [180–240]	180 [180–180]	180 [180–180]	180 [180–240]
All sizes				
Total no. of CBA	27	22	32	22
Occlusion grade 4	25 (93)	21 (96)	25 (78)	17 (77)
Time to –40ºC, s	30.7 ± 7.0	30.4 ± 3.4	29.8 ± 4.1	32.4 ± 4.8
Balloon nadir temperature, ºC	–58.0 ± 5.8	–55.1 ± 4.2	–58.4 ± 6.5	–57.1 ± 6.9
Thawing time to 0ºC, s	20.4 ± 5.9	18.8 ± 3.5	23.8 ± 9.3	22.8 ± 9.1
TTI, s	51 [36–71]	28 [24–41]	27 [24–50]	54 [32–65]
Single freeze success rate	15 (75)	18 (90)	14 (70)	16 (80)
31-mm balloon size only				
Total no. of CBA	20	20	22	19
Occlusion grade 4	19 (95)	19 (95)	18 (82)	15 (79)
Time to –40ºC, s	32.0 ± 7.6	30.2 ± 3.4	29.6 ± 3.6	32.8 ± 4.8
Balloon nadir temperature, ºC	–58 ± 6.3	–55.3 ± 4.3	–59.3 ± 6.6	–56.4 ± 6.8
Thawing time to 0ºC, s	20.8 ± 5.9	19.1 ± 3.5	25.4 ± 9.5	22.8 ± 9.9
TTI, s	55 [39–76]	30 [23–43]	27 [23–54]	53 [32–60]
28-mm balloon size only∗				
Total no. of CBA	7	2	10	3
Occlusion grade 4	6 (86)	2 (100)	7 (70)	2 (100)
Time to –40ºC, s	27.1 ± 2.9	33 (30 – 35)	30.0 ± 5.3	29.7 ± 4.5
Balloon nadir temperature, ºC	–57.9 ± 4.6	–53	–56.4 ± 6.2	–61.0 ± 7.0
Thawing time to 0ºC, s	19.3 ± 6.1	16 (13 – 19)	19.4 ± 4.2	23.0 ± 2.0
TTI, s	36 [28–51]	—	30 [24–50]	—

Continuous values given as mean ± SD or median [first–third quartile]. Other values given as n (%) unless otherwise indicated.

CBA = cryoballoon ablation; LIPV = left inferior pulmonary vein; LSPV = left superior pulmonary vein; RIPV = right inferior pulmonary vein; RSPV = right superior pulmonary vein; TTI = time to isolation.

∗ The 28-mm balloon size was used as an additional application if the 31-mm balloon size did not achieve isolation.

When comparing the grade 4 occlusion grade between the 28-mm and 31-mm balloon sizes, there was a trend toward better occlusion with the 28-mm balloon size in the RSPV compared to the 31-mm balloon size (90% vs 75%, *P* = .08) (Figure 2). The grade 4 occlusion rate was similar in other PVs. In total, the grade 4 occlusion grade of all PVs with the 31-mm balloon size was 86% (Figure 2). Addition of the 31-mm balloon size did not improve the grade 4 occlusion rate in comparison to only the 28-mm balloon size.

### UHDx mapping

Pre- and postablation mapping was successful in all patients. The results are given in Table 3 and Figure 3. Approximately one-third (35.4% ± 13.8%) of the LA posterior wall surface area was ablated after ablation with the 31-mm balloon size. ISA (true antral lesion area/[true antral lesion area + nonablated posterior wall surface are]) was 68.7% ± 8.5%. There was a significant reduction in the superior, middle, and inferior lines. The posterior antral lesion area per PV ranged from 1.4 ± 0.5 cm^2^ to 1.8 ± 0.6 cm^2^.

**Table 3:** Ultrahigh-density metrics

Variable	Preablation map (n = 20)	Postablation map (n = 20)	*P* value
LA posterior wall surface area, cm^2^	14.5 ± 2.7	9.4 ± 2.5	<.001
LA posterior wall ablated, %		35.4 ± 13.8	
Superior line, mm	36.8 ± 7.4	23.2 ± 8.7	<.001
Middle line, mm	39.4 ± 6.8	24.3 ± 7.0	<.001
Inferior line, mm	40.1 ± 7.8	29.4 ± 7.7	<.001
LPV ostia total, cm^2^		4.1 ± 0.8	
LSPV ostium, cm^2^		1.9 ± 0.4	
LIPV ostium, cm^2^		2.2 ± 0.7	
RPV ostia total, cm^2^		6.0 ± 1.6	
RSPV ostium, cm^2^		3.3 ± 1.0	
RIPV ostium, cm^2^		2.8 ± 1.0	
Total lateral circumferential antral lesion area, cm^2^		12.1 ± 2.0	
True lateral circumferential antral lesion area (ostia excluded), cm^2^		8.0 ± 2.1	
Total septal circumferential antral lesion area, cm^2^		19.1 ± 4.7	
True septal circumferential antral lesion area (ostia excluded), cm^2^		13.1 ± 3.5	
ISA, %		68.7 ± 8.5	
Posterior PV antral isolation area:			
LSPV, cm^2^		1.4 ± 0.5	
LIPV, cm^2^		1.8 ± 0.6	
RSPV, cm^2^		1.7 ± 0.7	
RIPV, cm^2^		1.7 ± 0.7	

Continuous values given as mean ± SD.

ISA = isolated surface area; LA = left atrium; LPV = left pulmonary vein; PV = pulmonary vein; RPV = right pulmonary vein; other abbreviations as in Table 2.

In 1 patient, inadvertent overlapping antral lesions resulted in a roofline with bidirectional block (Figure 4A). The preablation superior line in this patient was only 18 mm. There was a statistically significant linear correlation between the pre- and postablation superior line (Pearson correlation coefficient 0.66, 95% confidence interval 0.30–0.85, *P* = .002) (Figure 4B). The lower boundary of the 95% confidence interval transects the 10-mm postablation superior line at a preablation superior line of 27 mm. This implies that cryoablation with the 31-mm balloon size will result in a minimum postablation superior line of 10 mm when the preablation superior line is ≥27 mm.

### Safety

Regarding procedural safety, no vascular access complications, air embolism, pericardial effusion/tamponade, persistent PNP, or strokes/transient ischemic attacks were observed. One patient had a major in-hospital complication, an esophageal perforation that was discovered 1 day after the procedure. The complication was most likely due to use of the transesophageal probe and required surgery to correct the defect. During the CB procedure, this patient had a single freeze procedure of all PVs with the following nadir temperatures: LSPV –57°C (240 seconds); LIPV –56°C (180 seconds); RIPV –62°C (240 seconds); and RSPV –65°C (180 seconds).

### Clinical outcome

During a mean follow-up of 271 ± 95 days, 5 patients (25%) experienced an atrial tachyarrhythmia episode (2 AF, 2 atrial flutter, 1 supraventricular tachycardia). Three patients (15%) underwent a repeat procedure during follow-up. The first patient with supraventricular tachycardia underwent a slow pathway ablation for atrioventricular nodal reentrant tachycardia. The second patient with an atrial flutter had a redo PVI (all PVs were reconnected) and a cavotricuspid isthmus ablation. The third patient with recurrent AF had durable PVI and underwent a box isolation. Thus, the durable PVI rate was 50% (4/8 PVs) based on a very limited number of patients (n = 2).

## Discussion

The present study evaluated the acute antral lesion size after cryoablation with a 31-mm balloon size of a novel size-adjustable CB catheter in patients with paroxysmal AF. When using the 31-mm balloon size, large antral lesions can be made, resulting in ablation of approximately one-third of the posterior wall. In some patients, these large antral lesions may result in an inadvertent roof line. Although the smaller 28-mm balloon size may provide better PV occlusion in the RSPV, the single freeze isolation rate of the 31-mm balloon size is high (79% on PV level, 70% in the RSPV). The impact of these large antral lesions on long-term clinical outcome remains to be determined.

### Clinical performance of POLARx FIT

The clinical experience with the novel size-adjustable POLARx FIT is limited.^12^, ^13^, ^14^, ^15^, ^16^, ^17^, ^18^, ^19^ The first experience was in the FIT extension arm of the multicenter FROZEN-AF (Safety and Effectiveness IDE trial for Boston Scientific's Cryoballoon in the Treatment of Symptomatic Drug Refractory Paroxysmal Atrial Fibrillation) study, which demonstrated the safety and effectiveness of the POLARx FIT catheter in 50 patients.^12^ These results were corroborated in a large Japanese multicenter observational registry of 535 patients in whom the size-adjustable CB was associated with high procedural efficacy and safety. The acute PVI rate was 97.2%, and the single freeze isolation rate was 83.2%. Superior PVs (LSPV 76%, RSPV 83%) were more likely to be isolated by the 31-mm balloon size than the inferior PVs (LIPV 55%, RIPV 43%), reflecting better occlusion of the inferior PVs with the smaller 28-mm balloon size. The 1-year freedom from AF was 81.4% in a population in which 64% had paroxysmal AF and 36% (long-standing) persistent AF.

Use of the 31-mm balloon size in the different studies ranged from 17%–64% of all applications.^12^, ^13^, ^14^, ^15^, ^16^, ^17^, ^18^, ^19^ In 1 single-center prospective study, the 31-mm balloon size was used only if the 28-mm balloon size achieved suboptimal occlusion.^19^ This was the case in almost 1 of 5 PVs (17%) in this study. Our study demonstrated that a grade 4 occlusion could be achieved with the 31-mm balloon size in most PVs (86%), although complete occlusion was more difficult to achieve in the right-sided PVs. This could be related to the more compliant and smaller balloon size of the 28-mm balloon size. A more compliant balloon theoretically will provide better occlusion in oval shaped PVs. Furthermore, a smaller and more tubular shaped balloon will sit more deeply in the PV and provide better occlusion than a bigger balloon with a flatter anterior surface. Interestingly, in our study the complete occlusion rate did not improve with the addition of the 31-mm balloon size in comparison to the 28-mm balloon size.

Another potential benefit of a larger balloon size is a more antral position of the CB, theoretically resulting in less PNP.^20^ However, several studies have shown that the risk of PNP is still present with the 31-mm balloon size (up to 6.4%), not only in the RSPV but also in the RIPV.^15^, ^16^, ^17^^,^^26^ This could be related to the better occlusion with a larger CB resulting in deeper cooling of the phrenic nerve. In our study, there were no cases of PNP. The CONTRAST-CRYO II trial (Characteristics of Two Different Cryoballoon Systems for Treatment of Paroxysmal Atrial Fibrillation II; UMIN000052500) will assess the incidence of PNP (primary endpoint) of the size-adjustable POLARx FIT vs the Arctic Front Advance Pro.^27^ This is multicenter, prospective, randomized controlled trial enrolling 214 patients with paroxysmal AF. Secondary endpoints include procedural success, chronic success through 12 months, procedure-related adverse events, biophysiological parameters during applications for each PV, total procedural and fluoroscopy times, level of PVI and isolation area, and probability of non-PV foci initiating AF.

### Acute antral lesion size with other single-shot technologies

Comparison of the antral lesion size between different technologies is hampered by differences in patient populations, mapping technologies, definition of PV ostium, and low-voltage cutoff values. Currently, only 1 study (n = 22) by Goto et al^14^ also systematically evaluated antral lesion area with the 31-mm POLARx FIT CB; however, they used 3 different mapping systems (CARTO3, EnSite X, Rhythmia). In this study, the distance between the PV orifice and ablation edge was larger on the roof and posterior segments (∼15 mm) but relatively smaller on the anterior segment near the PV ridge (<10 mm) in the lateral PVs.^14^ On the septal side, the distribution of antral lesion was more symmetrical (10–15 mm). In our study, there was a smaller lateral antral lesion area than septal antral lesion area (8.0 ± 2.1 cm^2^ vs 13.1 ± 3.5 cm^2^), which could be explained by less myocardial tissue in the area between the anterior–superior side of the LSPV and the LA ridge.

Data on the antral lesion size of the first-generation 28-mm POLARx CB are limited. Prospective multicenter data (n = 29, all paroxysmal AF) from the Italian CHARISMA (Catheter Ablation of Arrhythmias With High Density Mapping System in the Real World Practice) Registry demonstrated that 45% ± 6% of the posterior wall was ablated when the POLARx CB was used.^28^ In this study, the antral lesion area was determined by UHDx mapping using a cutoff of 0.5 mV (in contrast to 0.3 mV in our study). The remaining nonablated posterior wall surface area and superior line in the CHARISMA Registry were 12.5 cm^2^ and 30.1 mm, respectively. In contrast, the nonablated posterior wall surface area and superior line in our study were much smaller (9.4 ± 2.5 cm^2^ and 23.2 ± 8.7 mm, respectively), suggesting that the antral lesion size was larger with the 31-mm CB and/or that LA dimensions were larger in the CHARISMA Registry.

The acute antral lesion area with the second-generation 28-mm Arctic Front Advance (Medtronic) was prospectively assessed by Kenigsberg et al^29^ in 43 patients using the EnSite mapping system (low voltage cutoff 1.2 mV). The lateral and septal antral lesion areas were 11.4 ± 5.3 cm^2^ and 11.3 ± 5.2 cm^2^, respectively. In our study, the lateral and septal antral lesion areas were 8.0 ± 2.1 cm^2^ and 13.1 ± 3.5 cm^2^, respectively. A study by Perotta et al^30^ demonstrated that the ISA (relative size of the low-voltage area in relation to the whole antral surface area including the posterior wall) with the second-generation Arctic Front Advance CB was 65% ± 8% (in contrast to 69% ± 9% in our study).

The acute antral lesion area with the “single-shot” pulsed field ablation (PFA) system FARAPULSE (Boston Scientific) seems larger than with the 31-mm POLARx FIT CB catheter. Gunawardene et al^24^ evaluated the antral lesion size by FARAPULSE in 20 patients using a similar method as in our study. Their total lateral and septal antral lesion areas (including PV ostia) were 20.5 ± 3.8 cm^2^ and 25.5 ± 5.7 cm^2^, respectively. In our study, the total lateral and septal antral lesion areas (including PV ostia) were 12.1 ± 2.0 cm^2^ and 19.1 ± 4.7 cm^2^, respectively. Also, other antral lesion area metrics such as the posterior PV antral isolation areas were larger with FARAPULSE.

It is important to stress that acute antral lesions with CB ablation may overestimate the size of durable antral lesions. This is reflected by PV reconnection after CB ablation but also lesion regression during a repeat ablation.^31^, ^32^, ^33^ This phenomenon not only is limited to thermal ablation but also is true for ablation with PFA.^34^

### Clinical implications

The data demonstrate that the 31-mm POLARx FIT CB provides an acute antral lesion size that is larger than with both the 28-mm POLARx and the second-generation 28-mm Arctic Front Advance CB but is smaller than PFA with the FARAPULSE catheter. However, it is important to understand that a large antral lesion may result in a small gap on the roof, which may be proarrhythmogenic and create roof-dependent atrial flutters. Several studies have demonstrated the inadvertent creation of overlapping antral lesion on the roof.^14^ In our study, there was a correlation between the preablation superior line and the postablation superior line. When the preablation superior line is ≥27 mm, then the risk of a small gap (<10 mm) after ablation with the use of 31-mm CB in both superior PVs is low. This information is relevant because registries have shown that the 31-mm balloon size is used more often in the superior PVs than in the inferior PVs.^15^ It may be better to use the 28-mm balloon size in the superior PVs in smaller atria to prevent small corridors on the roof.

### Study limitations

The most important limitation of the current study is the lack of a comparator group. This limits strong conclusions regarding the increase in antral lesion area in comparison with the 28-mm POLARx CB. Comparison to previous studies is hampered by differences in mapping techniques and definition of antral lesion metrics. Furthermore, no conclusions can be drawn regarding PVI durability because we have only limited data regarding PVI durability. Finally, it is important to stress that we only present data on acute antral lesion after cryoablation, and this most likely overestimates the durable antral lesion size.

## Conclusion

Cryoablation with the 31-mm balloon size of a novel size-adjustable CB results in a large and homogeneous antral lesion. In small atria, there is the potential for leaving a small nonablated corridor on the roof when using the 31-mm balloon in both superior PVs, which may be proarrhythmogenic. The availability of 2 balloon sizes in 1 CB catheter provides more flexibility; however, whether this translates into improved safety and long-term effectiveness requires further investigation.
